# Effects of Four Weeks of β-Alanine Supplementation on Repeated Sprint Ability in Water Polo Players

**DOI:** 10.1371/journal.pone.0167968

**Published:** 2016-12-08

**Authors:** Gabriel Motta Pinheiro Brisola, Guilherme Giannini Artioli, Marcelo Papoti, Alessandro Moura Zagatto

**Affiliations:** 1 Post-Graduate Program in Movement Sciences, São Paulo State University (Unesp), Institute of Biosciences, Rio Claro, São Paulo, Brazil; 2 Laboratory of Physiology and Sport Performance (LAFIDE), Department of Physical Education, São Paulo State University (Unesp), School of Sciences, Bauru, São Paulo, Brazil; 3 Department of Sport Science, School of Science and Technology, Nottingham Trent University, Nottingham, United Kingdom; 4 School of Physical Education and Sport of Ribeirão Preto, *University of São Paulo*, São Paulo, Brazil; Universita degli Studi di Verona, ITALY

## Abstract

The purpose of this study was to investigate the effect of four weeks of β-alanine supplementation on repeated sprint ability in water polo players. Twenty-two male water polo players participated in the study, divided randomly into two homogeneous groups (placebo and β-alanine groups). The study design was double-blind, parallel and placebo controlled. Before and after the supplementation period (28 days), the athletes performed two specific repeated sprint ability tests interspaced by a 30-minute swimming test. Participants received 4.8g∙day^-1^ of the supplement (dextrose or β-alanine) on the first 10 days and 6.4g∙day^-1^ on the final 18 days. There was no significant group-time interaction for any variable. The qualitative inference for substantial changes demonstrated a *likely beneficial* effect in the β-alanine group (β-alanine vs placebo) for mean time (6.6±0.4s vs 6.7±0.4s; 81% *likely beneficial*), worst time (6.9±0.5s vs 7.1±0.5s; 78% *likely beneficial*) and total time (39.3±2.5s vs 40.4±2.5s; 81% *likely beneficial*) in the first repeated sprint ability set and for worst time (7.2±0.6s vs 7.5±0.6s; 57% *possible beneficial*) in the second repeated sprint ability set. Further, was found substantial change for total time for both repeated sprint ability tests (80.8±5.7s vs 83.4±5.6s; 52% *possible beneficial*). To conclude, four weeks of β-alanine supplementation had a *likely beneficial* effect in the first set of repeated sprint ability tests and a *possible beneficial effect* for worst time in the second set performed in a specific protocol in water polo players.

## Introduction

β-alanine supplementation has become an ergogenic strategy widely used by competitive athletes from different sports [1], since it increases (~65% after 4 weeks) the intramuscular content of carnosine (β-alanyl-L-histidine) [2], a cytoplasmic dipeptide abundantly found in human skeletal muscle [2]. The pKa of carnosine is ~6.8 [3], meaning that carnosine is an obligatory physical-chemical buffer acting within the intramuscular pH transit range from resting (pH ~7.1) to fatigue (pH ~6.5) during high-intensity exercises [4]. Although buffer chemical is the main physiological function of carnosine [2], other functions are also attributed to this dipeptide. Dutka et al. [5] showed that carnosine can act like a calcium regulator, increasing the sensitivity of the contractile apparatus to calcium ions (Ca^2+^). Furthermore, studies have suggested that carnosine can act as a combination of these functions, as a local “pump”, by exchanging Ca^2+^ for H^+^ [6, 7].

As a matter of fact, several recent studies have confirmed that β-alanine supplementation does improve buffering capacity [8, 9] and high-intensity exercise performance [8–11], especially in exercises where there is a high level of muscle acidosis [1]. Although the ergogenic effects of β-alanine have been investigated in various exercise models [12], much less is known about its potential use in different sport disciplines. In this regard, studies have examined the β-alanine ergogenic potential mainly in individual sports (i.e., running [11], swimming [10], judo [13] and others). The results, however, are mixed and the performance enhancing effects seem to be related to the specificities of each sport discipline.

In many team sports, repeated sprint ability (RSA) is a key-component to competitive success [14]. Given that repeated sprints interspersed by short resting intervals are known to cause large increases in blood [15] and muscle acidosis [4], it is plausible that the increase in muscle carnosine brought about by β-alanine supplementation could be of great ergogenic potential in many team sports. However, there is little experimental data addressing the ergogenic effects of β-alanine supplementation in these disciplines [16, 17]. Although some studies have investigated the effects of β-alanine supplementation in repeated sprint protocols [8, 18–20], no studies have examined its effects in sport-specific testing protocols performed in non-laboratory conditions. Thus, further investigations in this perspective are warranted, especially those involving well-trained athletes tested in sport-specific situations [1].

Water polo is characterized by high intensity intermittent efforts with athletes being required to perform repeated short maximal sprints (~6 s) followed by short periods of recovery (~10 s) [21] over a relatively long period of time (a match lasts ~32 minutes) [22]. As result, there is a high glycolytic demand in water polo, as indicated by the elevated blood lactate concentrations ([La^-^]; i.e., 7.7±1.0 mmol/L) [23], suggesting a high degree of acidosis

In fact, there is a clear decline in physical performance in the final quarter of a water polo match in comparison with the previous ones, especially in the time spent in high intensity efforts (from 18.1% to 12.6%) [21] and in physiological variables such as [La^-^] (~14%) [24]. Thus, the need for nutritional strategies capable of delaying fatigue in order to improve performance, particularly in the final moments of the match, becomes evident.

In this study, we investigated the effects of 4 weeks of β-alanine supplementation on a water polo-specific RSA testing protocol. We hypothesized that β-alanine would improve RSA, especially in the final moments of the protocol, when acidosis and fatigue limit performance.

## Materials and Methods

### Subjects

**Fig 1:**
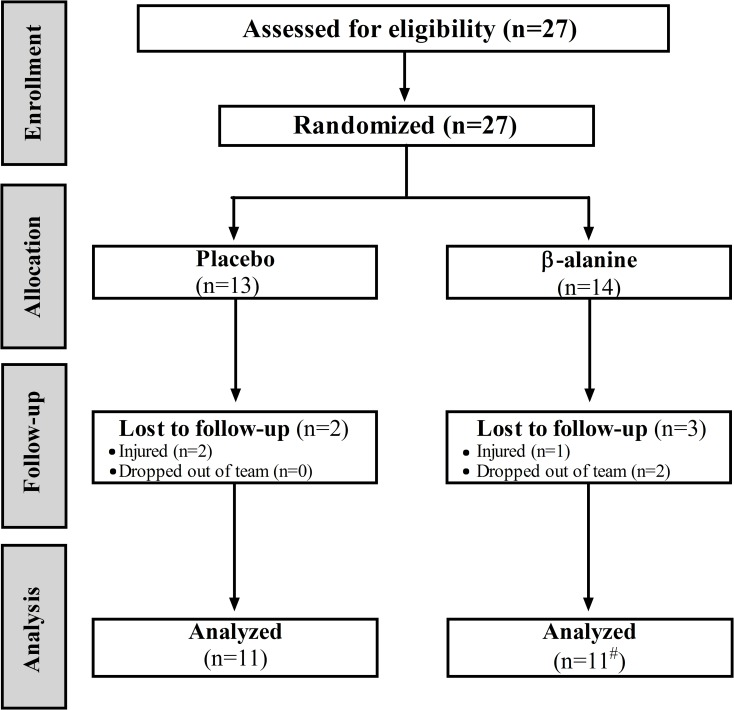
Schematic recruitment of participants and progression of each stage of the study. # 2 participants of the β-alanine group were excluded from part of the analysis. One stopped before crossing the finish line during the second set of RSA tests in the assessment and the other had a cramp during the second set of RSA tests in the reassessment. They were excluded only from the respective analyses.

Twenty–two well-trained male water polo athletes [mean±standard deviation (95% confidence interval): age = 18±4 (17 to 20) years, body weight = 78.5±9.5 (74.7 to 82.3) kg and height = 1.79±0.06 (176.1 to 181.3) m)] from a first division team participated in the study. Although 27 participants were recruited, 22 finished all tests, as presented in Fig 1. The athletes´ team won the elite national water polo championship in the year the study was conducted. All participants trained twice a day, six times per week. Training routines comprised resistance training sessions (strength, hypertrophy and power), lasting about one hour, and specific water polo training (swimming, technical, tactical and match simulations), lasting about three hours. The researchers did not interfere in the athletes´ training program.

The study began in the general preparatory phase (February, 2015) of the season and finished in the competitive phase (March, 2015). The weekly training loads during this period (measured by the session rating of perceived exertion [RPE-Session]; rating of perceived exertion x total session duration) [25] were 3723±902 arbitrary units, 3764±843 arbitrary units, 4494±1223 arbitrary units and 4522±1620 arbitrary units in the first, second, third and fourth week, respectively.

Participants did not take any dietary supplements for a minimum of 3 months prior to the start of the study. Before signing the informed consent form, the athletes were informed about the risks and benefits involved in participation. Informed consent was obtained from all participants included in the study and all procedures were conducted in accordance with the declaration of Helsinki and previously approved by the Ethics Committee of the São Paulo State University (Protocol number 430.916/2013). In case of participants under 18 years of age, the provided informed consent was signed by their parents.

### Experimental design

**Fig 2:**
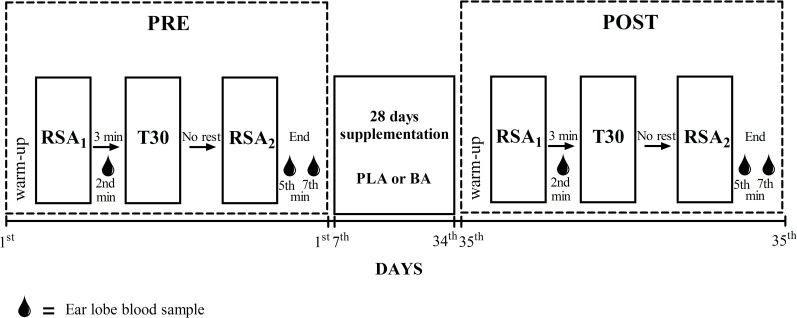
Time line and schematic of the tests. RSA_1_ = RSA test performed before T30; RSA_2_ = RSA test performed after T30; T30 = 30-minute swimming test.

The study was conducted in a randomized, double-blind, parallel-group, placebo-controlled manner. The experimental design is shown in Fig 2. Initially, the participants performed the water polo-specific RSA testing protocol (RSA test+30-minute swimming test + RSA test) and were then randomly allocated to receive either a placebo [PLA; age: 18±3 (17 to 20) yrs, body mass: 81.4±8.8 (76.3 to 86.1) kg, height: 1.79±0.07 (1.76 to 1.83) m and protein intake: 2.0±0.6 (1.6 to 2.4) g∙kg^-1^∙day^-1^) or β-alanine (BA; age: 19±5 (16 to 22) yrs, body mass: 75.7±9.6 (69.9to 80.9) kg, height: 1.79±0.06 (1.75 to 1.83) m and protein intake: 2.0±0.5 (1.8 to 2.4) g∙kg^-1^∙day^-1^]. Randomization was equalized by performance, athlete´s position and protein intake (measured by a 3-day food recall) to ensure homogeneity between groups. After the 28-day supplementation period, all participants were reassessed.

### Procedures

#### Repeated Sprint Ability Tests and 30-minute swimming test (T30)

All procedures were performed in the same period of the day during which the athletes´ training sessions routinely took place. All athletes were accustomed to performing repeated sprints as part of their regular training. The RSA and T30 tests were conducted in a 25-m indoor pool with a water temperature of 27±1°C.

The RSA test was performed twice, interspersed by a 30-minute swimming test (T30) [26]. The first RSA test (RSA_1_) was performed after a standard warm-up (the same as used before every training session). The T30 was performed 3min after the RSA_1_ and the second RSA (RSA_2_) was performed immediately after the T30 (Fig 2).

The RSA test consisted of 6 maximal sprints of 10 m interspersed by 17-s recovery intervals [27]. During the intervals, the participants essentially floated as they were not allowed to touch their hands on the edge of the pool. The RSA used in this study was based on real time-motion data of water polo matches, as described by Tan et al. [27] (Intraclass Correlation Coefficient = 0.93; coefficient of variation = 1.2%).

A 30 Hz digital camera (GoPro Hero 3+ Black, San Mateo, California, USA) was used to record the time of each sprint and the videos were later analyzed using the open-license software Kinovea (Kinovea 0.8.15 for Windows; available at http://www.kinovea.org/) [28]. The time to complete each sprint was used to determine the best time, mean time, worst time, total time and the total time for both RSA tests (i.e., RSA_1_+RSA_2_). Moreover, during the RSA tests the absolute decrement (Eq 1) and decrement percentage (Eq 2) were calculated as follows:

AbsoluteDecrement=Totaltime-Idealtime (Equation 1)

DecrementPercentage=[Totaltime÷Idealtime×100]-100 (Equation 2)

where the ideal time is the best time of sprints multiplied by 6 [29].

The 30-minute swimming test (T30) started 3 min after RSA_1_ (passive recovery) with the athletes being instructed to swim in freestyle the furthest possible distance in 30 minutes [26]. During the T30, the participants did not receive any feedback on the time elapsed. The test was also recorded using a digital camera (GoPro Hero 3+ Black, San Mateo, California, USA) and the total distance covered was determined by analyzing the video.

Blood samples (25 μL) were collected from the ear lobe for the determination of lactate concentration in the second minute after RSA_1_ and in the fifth and seventh minute after RSA_2_, allowing for the identification of peak lactate concentration ([La^-^]_peak_). The blood samples were analyzed using an electrochemical lactate analyzer (YSI 2300 STAT, Yellow Spring Instruments, Yellow Spring, Ohio, USA) (measurement error of ±2%).

#### Supplementation protocol

Participants received either β-alanine (99.9% pure β-alanine; CarnoSyn™, NAI, San Marcos, California, USA) or a placebo (dextrose, Neonutri, Poços de Caldas, Minas Gerais, Brazil) for 28 days. Both the placebo and β-alanine were packed in gastro-resistant capsules (hidroxipropilmetilcelulose, DrCaps^TM^, Capsugel, Puebla, Puebla, Mexico), identical in appearance. On the first 10 days, participants received 4.8g∙day^-1^, divided into six daily doses of 800mg. On the final 18 days, participants received doses corresponding to 6.4g∙day^-1^, divided into four daily doses of 1600mg. If a participant forgot to take a dose, they were required to take an extra dose in another period or on a different day to complete the total dose of 163.2g by the end of the study. All doses were taken with a minimum interval of two hours and preferably with meals to avoid paresthesia (flushing sensation on the skin) [2, 9]. The participants were individually monitored during the study regarding side-effects of supplementation (i.e., paresthesia) and were instructed not to comment about side-effects to other players.

#### Statistical analyses

The calculation of sample size (G*Power 3.1) was based on the effect size (ES) of percentage of changes (Δ%) in 200-m swimming performance, reported in the study of De Salles Painelli et al. [10]. Thus, the minimum sample size required for the study considering an alpha of two-sided of 0.05 and a beta of 0.85 for unpaired *t* test (i.e. Δ placebo × Δ β-alanine) was twenty-two participants. All data were checked for normality using the Shapiro-Wilk test and are presented as means±standard deviation and 95% confidence interval (CI95%). In order to compare the outcomes, between-subject factor (placebo vs. β-alanine) and within-subject factor 'time' (pre and post), a two-way repeated measure ANOVA was used for all variables. In addition, the Mauchly’s test of sphericity was applied and the Greenhouse-Geisser Epsilon correction was used when the sphericity criteria was not met. The analyses were completed with the Bonferroni *post hoc*. The unpaired *t* test was used for comparing relative changes (Δ%) between groups. In all cases, a 5% significance level was considered and the data were analyzed in SPSS version 15.0 for Windows (SPSS Inc., Chicago, Illinois, USA).

In addition to conventional statistical analysis, magnitude-based inference analysis was used [30]. The raw outcomes were log-transformed before analysis to reduce non-uniformity of error [31].The values are expressed as standardized mean differences (Cohen’s *d*) [32] and the threshold values for Cohen’s *d* statistical power were considered as >0.2 (small), >0.5 (moderate), and >0.8 (large). The chances of the effect being positive, trivial or negative were calculated based on the smallest worthwhile change (SWC; 0.2 multiplied by the between-subject standard deviation). If the probabilities of the effect being substantially positive and negative were both >5%, the effect was reported as *unclear*, otherwise the effect was clear. Thus, the changes were qualitatively evaluated as follows: <1% = *most unlikely*; 1%–5% = *very unlikely*; 5%–25% = *unlikely*; 25%–75% = *possibly*; 75%–95% = *likely*; 95%–99% = *very likely*; and >99% = *most likely* [31].

## Results

Few participants reported paresthesia during the study, of which 3 were in the β-alanine group and 1 in the placebo group.

**Fig 3:**
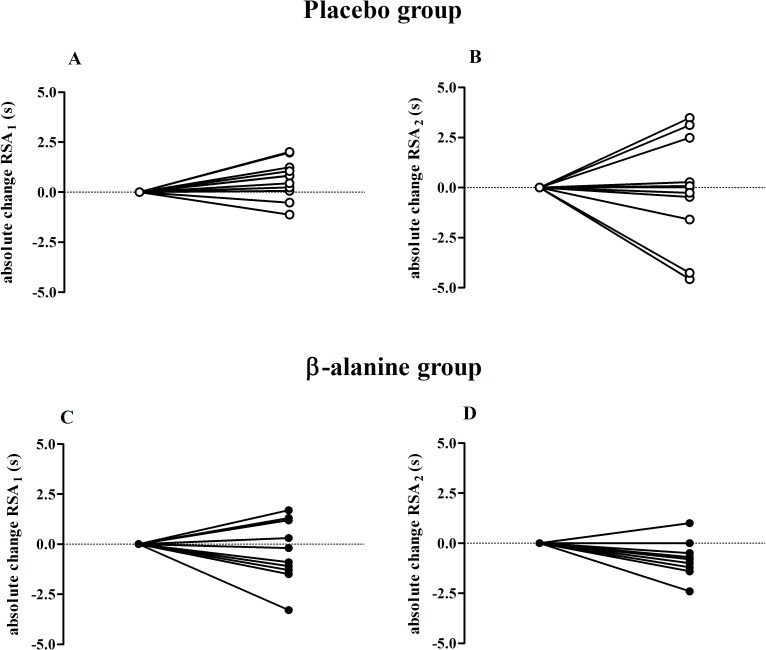
Individual absolute change (post-pre) for the placebo and β-alanine groups in RSA testing. A = Individual absolute change in the placebo group in RSA_1;_ B = Individual absolute change in placebo group in RSA_2;_ C = Individual absolute change in the β-alanine group in RSA_1;_ D = Individual absolute change in the β-alanine group in RSA_2._ Note: 2 participants in the β-alanine group were excluded from the RSA_2_ analysis, as shown in Fig 1.

All performance results are shown in Table 1. There was no significant group-time interaction for any variable, however, there was a significant time effect in [La^-^] for RSA_1_ in both groups (*F* = 48.7; *p*<0.001; *post hoc*: Placebo and β-alanine groups *p*<0.001;statistical power = 100%) and in [La^-^]_peak_ for RSA_2_ only in placebo group (*F* = 10.9; *p =* 0.004; *post hoc*: Placebo group *p* = 0.006; statistical power = 88%). Furthermore, both groups significantly improved performance in the T30 after the supplementation period (*F* = 35.3; *p<*0.001; *post hoc*: Placebo group *p*<0.001; β-alanine group *p* = 0.001; statistical power = 100%). The individual changes for total time in the RSA testing are shown in Fig 3 where it is possible to verify an effect of β-alanine on RSA_2,_ with 7 athletes out of 11 improving their individual performances (S1 Dataset**)**.

**Table 1:** Between-subject factor and within-subject factor comparison of water polo-specific RSA testing protocol.

	Placebo Group			β-alanine Group		
	Pre Supplementation	Post Supplementation	Δ%	Pre Supplementation	Post Supplementation	Δ%
***RSA*_*1*_**						
**Best Time (s)**	6.3±0.5 (6.0 to 6.5)	6.3±0.4 (6.1 to 6.5)	+0.7	6.3±0.3 (6.1 to 6.5)	6.3±0.4 (6.0 to 6.5)	-0.5
**Mean Time (s)**	6.6±0.4 (6.4 to 6.9)	6.7±0.4 (6.5 to 7.0)	+1.6	6.6±0.4 (6.4 to 6.8)	6.6±0.4 (6.3 to 6.8)	-0.9
**Worst Time (s)**	6.9±0.5 (6.7 to 7.2)	7.1±0.5 (6.8 to 7.3)	+1.9	6.9±0.4 (6.7 to 7.1)	6.9±0.5 (6.6 to 7.1)	-0.8
**Total Time (s)**	39.7±2.7 (38.2 to 41.2)	40.4±2.5 (38.9 to 41.8)	+1.6	39.7±2.3 (38.4 to 41.0)	39.3±2.5 (37.9 to 40.8)	-0.9
**Absolute Decrement (s)**	2.0±0.8 (1.6 to 2.5)	2.4±1.1 (1.8 to 3.0)	+18.7	1.9±0.6 (1.6 to 2.3)	1.8±0.6 (1.5 to 2.1)	-7.6
**Decrement Percentage (%)**	5.4±2.3 (4.2 to 6.8)	6.3±2.8 (4.9 to 8.0)	+16.3	5.1±1.4 (4.3 to 5.9)	4.7±1.4 (4.0 to 5.5)	-7.0
**[La^-^] (mmol/L)**	7.4±1.7 (6.4 to 8.3)	4.9±1.1 (4.2 to 5.5)*	-33.9	6.9±1.4 (6.0 to 7.6)	4.9±1.8 (3.9 to 5.9)*	-29.9
**T30 (m)**	1852±204 (1741 to 1970)	1938±229 (1805 to 2073)*	+4.6	1957±159 (1877 to 2054)	2020±165 (1930 to 2123)*	+3.3
***RSA*_*2*_**						
**Best Time (s)**	6.9±0.5 (6.6 to 7.2)	6.8±0.4 (6.6 to 7.0)	-1.8	6.8±0.4 (6.6 to 7.1)	6.6±0.5 (6.3 to 6.9)	-3.7
**Mean Time (s)**	7.2±0.6 (6.9 to 7.5)	7.2±0.6 (6.9 to 7.5)	-0.4	7.1±0.5 (6.9 to 7.4)	6.9±0.5 (6.6 to 7.2)	-3.5
**Worst Time (s)**	7.5±0.6 (7.1 to 7.9)	7.5±0.6 (7.2 to 7.9)	+0.1	7.5±0.6 (7.2 to 7.9)	7.2±0.6 (6.8 to 7.5)	-4.2
**Total Time (s)**	43.2±3.4 (41.2 to 45.0)	43.1±3.3 (41.3 to 44.9)	-0.4	42.8±2.9 (41.2 to 44.5)	41.3±3.2 (39.4 to 43.2)	-3.5
**Absolute Decrement (s)**	1.8±0.9 (1.3 to 2.3)	2.4±1.3 (1.7 to 3.1)	+33.4	1.9±1.3 (1.2 to 2.7)	2.0±0.9 (1.5 to 2.5)	+2.6
**Decrement Percentage (%)**	4.3±2.1 (3.1 to 5.5)	5.8±3.0 (4.3 to 7.6)	+35.0	4.8±3.2 (3.0 to 6.8)	5.1±2.3 (3.8 to 6.5)	+5.6
**Total Time for both RSA test (s)**	82.9±5.9 (79.4 to 86.1)	83.4±5.6 (80.3 to 86.5)	+0.6	82.7±5.0 (79.7 to 85.5)	80.8±5.7 (77.4 to 84.3)	-2.5
**[La^-^]_peak_ (mmol/L)**	5.4±1.8 (4.3 to 6.4)	3.9±1.7 (3.0 to 4.9)*	-26.7	5.8±1.8 (4.8 to 6.9)	5.1±2.1 (3.9 to 6.3)	-13.0

Values expressed as mean ± standard deviation (95% confidence interval). RSA_1_ = RSA test performed before T30; RSA_2_ = RSA test performed after T30; T30 = 30-minute swimming test; [La^-^] = lactate concentration; [La^-^]_peak =_ lactate concentration peak; Δ % = percentage difference of mean.

*Significant difference from pre (*p*≤0.05).

**Fig 4:**
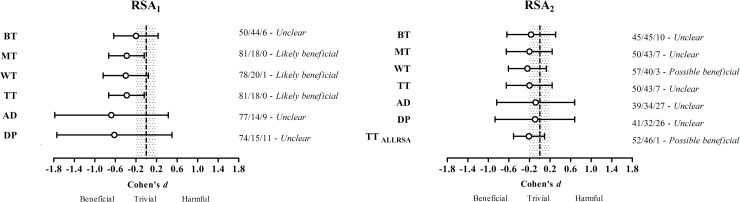
Magnitude-based inference analysis between-groups. Trivial area (grey) threshold is -0.2 to 0.2 and bars indicate ±90% confidence limits. AD = absolute decrement; BT = best time; DP = decrement percentage; MT = mean time; RSA_1_ = RSA test performed before T30; RSA_2_ = RSA test performed after T30; TT = total time; TT _ALLRSA =_ total time for both RSA tests; WT = worst time.

The between-group changes analyzed by magnitude-based inference are presented in Fig 4. In RSA_1_, the β-alanine group showed a *likely beneficial* effect for mean time, worst time and total time. In RSA_2_ all effects were *unclear*, except for worst time and total sprint time for both RSA, where β-alanine presented a *possibly beneficial* effect. The between-group effect for T30 in Cohen’s *d* units was *trivial* (2/88/10 for beneficial, trivial and harmful, respectively).

## Discussion

The main finding of the present study was that 4 weeks of β-alanine supplementation did not significantly improve RSA evaluated in a water polo-specific testing protocol. However, the magnitude-based inference analyses showed *likely beneficial* effects for β-alanine supplementation in comparison to the placebo treatment for mean time, worst time and total time in the RSA performed before T30 (RSA_1_) and *possible beneficial* effects for worst time in the RSA performed after T30 (RSA_2_) and for total time for both RSA tests. Thus, according to our findings, it is plausible to assume that 4 weeks of β-alanine supplementation can only slightly improve RSA in well-trained water polo players. However, it is important to emphasize that the ergogenic effect of β-alanine supplementation is more likely on first set of RSA test performed on a water polo-specific protocol.

Chronic β-alanine supplementation is a nutritional strategy that has been used to improve performance in some sports, especially those characterized by high intensity [10, 11, 13]. The ergogenic effect of β-alanine supplementation is related to an increase in carnosine content, which is a dipeptide that has multiple functions [33], mainly muscle buffering [2].Several studies have shown that carnosine content can increase expressively after 4 weeks of β-alanine supplementation. Harris et al. [10] showed that after 4 weeks with daily doses of 3.2 g∙day^-1^ and 6.4 g∙day^-1^of β-alanine the carnosine content increased by 42.1% and 64.2%, respectively. Similarly, Hill et al. [9] demonstrated an increase in carnosine content of 58.8% after 4 weeks of β-alanine supplementation with daily doses ranging from 4.0 g∙day^-1^ to 6.4 g∙day^-1^. Furthermore, recently Bex et al. [34] showed that carnosine loading with chronic β-alanine is more pronounced in trained (cyclists, swimmers and kayakers) than compared with untrained muscles. Thus, although the present study did not measure intramuscular carnosine content, which is assumed as a study limitation, it is plausible that 4 weeks of β-alanine supplementation with doses of 4.8 g∙day^-1^ to 6.4 g∙day^-1^ would be effective to increase the carnosine content in water polo athletes.

Our initial hypothesis that β-alanine would improve RSA in the final moments of the protocol cannot be fully confirmed. The RSA_2_ was carried out in order to mimic the demand during a water polo match, in which athletes perform repeated sprints intermittently [21]. Any improvement in the RSA_2_ could represent an improvement in some important actions, particularly in the latter quarters of a water polo match (i.e., 3^rd^ and 4^th^), during which a decline in physical performance is usually observed [21, 23]. However, β-alanine seems to be unable to promote substantial improvements in RSA under these conditions, since only worst time presented a *possible beneficial* (57%) effect through magnitude-based inference analysis. In addition, although Fig 3 demonstrates a slight trend in the β-alanine group to improve total time and the Δ% improved ~3% for time variables in the β-alanine group, whereas in the placebo group the improvement was only ~0.5% (Table 1), these differences (between-subject factor and within-subject factor) were very small and widely dispersed, making them non-significant. Thus, the findings in RSA_2_ shown in the present study were in accordance with Saunders et al. [19], who investigated the effects of β-alanine supplementation on RSA evaluated after efforts that simulated the physical demands of a football match and also did not find improvements by conventional statistical analysis.

When the RSA test was performed without previous efforts (i.e., RSA_1_), β-alanine supplementation presented greater positive results than RSA_2_. However, these positive results were again found only in magnitude-based inference analysis, while in conventional statistical analysis no positive results were found. The magnitude-based inference analysis showed a *likely beneficial* effect of β-alanine supplementation for mean time, worst time and total time compared with the placebo treatment (Fig 4). Although the improvements in RSA_1_ were found only in magnitude-based inference analysis, they could have some importance for the sport modality. Magnitude-based inference analysis is a statistical approach used to appropriately deal with the importance of an effect in the real-world [31]; it has been widely used in studies with sports performance [10, 11, 18] in which small differences can be representative. Thus, it is plausible to assume a slight improvement in RSA_1_, which is considered a key-component for competitive success in team sports[14], including water polo [21]. In water polo matches, sprints are performed to gain an advantage over the opponent, perform counterattacks and defend against attacking [22]; and often occur repetitively [21], highlighting the importance of RSA for water polo.

These RSA_1_ results are similar to other studies that investigated the ergogenic effects of β-alanine supplementation on RSA [8, 18–20] and also did not find significant interactions using conventional statistical analysis. On the other hand, the present study was the first to report positive outcomes, through magnitude-based inference analysis, of β-alanine supplementation on RSA. Ducker et al. [18] and Saunders et al. [19] despite using this statistical approach, which is ideal for detecting small changes in sports performance, did not find meaningful effects of β-alanine supplementation on RSA.

Since RSA is highly dependent on the buffering capacity of H^+^ ions [15], more expressive results were expected for the ergogenic effects of β-alanine supplementation. Repeated efforts cause large reductions in muscle pH [15] and may result in strength decrements [35], inhibition of creatine phosphate resynthesis [36], inhibition of glycolysis [37], impairment in the functioning of the muscle contractile process [38] and consequently decreases in performance [12]. Therefore, since β-alanine supplementation can increase the buffering capacity, it could result in a delay in these processes that may impair RSA [1], but the beneficial effects found in the present study were discreet.

The [La^-^] in both RSA tests (RSA_1_and RSA_2_) was not influenced by β-alanine supplementation. After the supplementation period, both groups (placebo and β-alanine) showed significant reductions in [La^-^] in RSA_1_ compared to the pre-supplementation moment, while in RSA_2_ only the placebo group showed a significant reduction in [La^-^]_peak_. However, although not statistically significant, the reduction in [La^-^]_peak_ was also evident in the β-alanine group (Table 1) compared to the pre-supplementation period. These reductions may have been due to an improvement in the oxidative pathway of the team generated by training, since both groups demonstrated improvement in T30. Thus, it is likely that an improvement in the oxidative pathway was responsible for increased lactate removal from the efforts, with a consequent reduction in final [La^-^] [39]. A more accurate explanation could be given if the intramuscular and blood pH had been analyzed after efforts in the present study, which could better represent the magnitude of H^+^ ion accumulation.

Another possible limitation of the present study was that three participants reported paresthesia symptoms after β-alanine supplementation, which may have compromised the blinding of these participants to the supplementation condition. Despite of recommendation to the players ingest the supplements during the meals and with intervals minimum of two hours between doses, and the supplements packed in gastro-resistant capsules to avoid the occurrence of paresthesia, probably the high acute dose of β-alanine (1600 mg) may have caused the side effect. Thus, in future studies will be advised to use a lower (but longer) supplementation dose and/or use slow-release tablets [40].

## Conclusion

Therefore, according to the findings of the present study, 4 weeks of β-alanine supplementation did not significantly improve RSA evaluated in a water polo-specific testing protocol. However, the *likely beneficial* effect shown by magnitude-based inference analyses, on mean time, worst time and total time in the first set of RSA testing (RSA_1_) suggest a slight improvement in RSA_1._ Thus, the practical applications of the present study are that 4 weeks of β-alanine supplementation could slightly improve RSA in the first quarter of a water polo match, but not necessarily in the later quarters. Since water polo sprints are performed to gain an advantage over the opponent, perform counterattacks and defend against attacking [22], and often occur repetitively, these slight improvements could be of some importance for water polo players. However, to further state the effects of β-alanine supplementation on water polo performance, future research should be conducted tracking water polo players in simulated games.

## Supporting Information

S1 Dataset Individual data from study. (XLSX)
